# Investigations on the high performance of InGaN red micro-LEDs with single quantum well for visible light communication applications

**DOI:** 10.1186/s11671-023-03871-z

**Published:** 2023-07-27

**Authors:** Fu-He Hsiao, Tzu-Yi Lee, Wen-Chien Miao, Yi-Hua Pai, Daisuke Iida, Chun-Liang Lin, Fang-Chung Chen, Chi-Wai Chow, Chien-Chung Lin, Ray-Hua Horng, Jr-Hau He, Kazuhiro Ohkawa, Yu-Heng Hong, Chiao-Yun Chang, Hao-Chung Kuo

**Affiliations:** 1 Semiconductor Research Center, Hon Hai Research Institute, Taipei, 11492 Taiwan; 2 Department of Electrophysics, College of Science, National Yang Ming Chiao Tung University, Hsinchu, 30010 Taiwan; 3 Department of Photonics and Institute of Electro-Optical Engineering, College of Electrical and Computer Engineering, National Yang Ming Chiao Tung University, Hsinchu, 30010 Taiwan; 4 Computer, Electrical and Mathematical Sciences and Engineering (CEMSE) Division, King Abdullah University of Science and Technology (KAUST), Thuwal, 23955 6900 Saudi Arabia; 5 Department of Electrical Engineering, National Taiwan University, Taipei, 10639 Taiwan; 6 Institute of Electronics, National Yang Ming Chiao Tung University, Hsinchu, 30010 Taiwan; 7 Department of Materials Science and Engineering, City University of Hong Kong, Kowloon, 999077 Hong Kong Special Administrative Region China; 8 Department of Electrical Engineering, National Taiwan Ocean University, Keelung, 202301 Taiwan

**Keywords:** Micro-LED, Red InGaN-based LED, Visible light communication

## Abstract

In this study, we have demonstrated the potential of InGaN-based red micro-LEDs with single quantum well (SQW) structure for visible light communication applications. Our findings indicate the SQW sample has a better crystal quality, with high-purity emission, a narrower full width at half maximum, and higher internal quantum efficiency, compared to InGaN red micro-LED with a double quantum wells (DQWs) structure. The InGaN red micro-LED with SQW structure exhibits a higher maximum external quantum efficiency of 5.95% and experiences less blueshift as the current density increases when compared to the DQWs device. Furthermore, the SQW device has a superior modulation bandwidth of 424 MHz with a data transmission rate of 800 Mbit/s at an injection current density of 2000 A/cm^2^. These results demonstrate that InGaN-based SQW red micro-LEDs hold great promise for realizing full-color micro-display and visible light communication applications.

## Introduction

So far, InGaN-based light-emitting diodes (LEDs) have found their way into a broad range of applications in our everyday lives. In recent years, emerging technologies such as 5G, artificial intelligence, image recognition technology, and augmented reality (AR)/virtual reality (VR) have rapidly developed with the concept of the metaverse and increasing demand for entertainment experiences and life efficiency. Given their high resolution, low power usage, high brightness, and quick response time, micro-LEDs stand as the most promising technology for future displays and visible light communication (VLC) systems [1]. Assembling red, green, and blue (RGB) micro-LEDs to achieve full-color miniature displays or VLC systems is a feasible approach. InGaN-based blue and green micro-LEDs have been investigated by many teams to maintain excellent performance with their shrinking size [2, 3]. When it comes to red LEDs, AlGaInP-based red LEDs were once the first choice due to their high efficiency. However, due to their poor thermal stability and a significant decrease in size-dependent quantum efficiency with increasing temperature, they are no longer preferred [4–6]. The InGaN-based red micro-LEDs have the advantage of utilizing the same material as green and blue micro-LEDs, which lowers process costs. Their efficiency remains less impacted by size reduction and high temperatures, positioning them as key contenders for miniaturization to the micrometer scale [7, 8]. However, InGaN-based red micro-LEDs still present significant challenges in terms of external quantum efficiency (EQE) owing to the presence of piezoelectric fields in InGaN strained quantum wells. These fields result in the manifestation of severe quantum-confined Stark effects (QCSE) [9–11]. By optimizing the substrate, it is possible to alleviate the strain on the quantum wells [12, 13]. Recently, efforts have been undertaken to develop high-efficiency InGaN-based red micro-LEDs. Pasayat et al. [14] reported an on-wafer EQE of 0.2% for 632 nm InGaN red micro-LEDs with ultrasmall 6 μm × 6 μm sizes. By thickening the GaN buffer layer to suppress the remaining in-plane stress, Iida et al. [15] demonstrated an InGaN-based red LED operating at 633 nm, which exhibited an EQE of 1.6% with a direct current injection of 20 mA. Chan et al. [16] demonstrated 633-nm InGaN red LEDs, which were deposited on a relaxed InGaN/GaN superlattice buffer, with a low forward voltage and a high growth temperature in the active region. Iida et al. [17] achieved an EQE of 4.3% at 10.1 A/cm^2^ for a 621-nm InGaN-based single quantum well (SQW) LED. Pandey et al. [18] optimized the Mg doping in the p-GaN layer to demonstrate an SQW red-emitting sub-micrometer scale LED with an EQE of 8.3%.

Wireless communication has a promising new technology known as VLC that boasts higher bandwidth and security compared to radio frequency (RF). VLC is suitable for a variety of communication environments, making it the most promising technology for high-speed communication soon [19–22]. Micro-LEDs are particularly advantageous for high-speed visible communication applications due to their reduced junction capacitance resulting from their small size, in contrast to conventional LEDs with narrower modulation bandwidth [23–25]. Therefore, integrating InGaN-based RGB micro-LEDs on the same platform holds great promise for next-generation full-color VLC technology. However, several challenges need to be addressed to achieve maximum modulation bandwidth and high-speed VLC, including the need for efficient current injection, high-quality epitaxial growth, and high-speed modulation. The exceptional performance of InGaN-based blue and green micro-LEDs in efficiency and modulation bandwidth has been demonstrated by various research teams. For instance, Lin et al. [26] showed that a 2 × 2 green micro-LED array with an emitting aperture size of 50 μm could support optical wireless communication with data rates exceeding 1.5 Gbit/s. Xu et al. designed ultrathin InGaN QW structures with increased In doping and localization effects to minimize polarization field using optimized growth techniques. This shortened the carrier lifetime and increased the tuning bandwidth of their *c*-plane InGaN/GaN blue micro-LED, achieving a high − 3 dB bandwidth of 1.53 GHz, making it ideal for high-speed applications [27]. The same team designed and fabricated the semi-polar green micro-LEDs with a high − 3 dB bandwidth of 1.035 GHz due to the reduced QCSE [28]. In 2022, we reported an InGaN red micro-LED with an EQE of 5%, modulation bandwidth of 271 MHz, and transmission rate of 350 Mbit/s at 2000 A/cm^2^ [29]. Even though InGaN red micro-LEDs have become a hot research topic, there is a need to improve the maximum bandwidth and fastest speed of red micro-LEDs to realize high-performance RGB micro-LED VLC systems. Additionally, the number of QW may affect the modulation bandwidth, as shown by Lan et al. and Cai et al., who suggested that a reduced number of QWs yielded the best response in terms of tuning characteristics [30, 31].

We conducted a study to improve the modulation bandwidth and speed of red micro-LEDs for potential use in VLC. We compared InGaN-based micro-LEDs with SQW and double quantum wells (DQWs) structures to identify the ideal structural and optical properties for epitaxial growth of efficient InGaN-based LEDs with varying numbers of QWs on *c*-planar patterned sapphire substrates. Our investigation revealed that the InGaN-based SQW red LEDs possess higher internal quantum efficiency (IQE) and shorter carrier lifetime, indicating their superior performance in realizing efficient red micro-LEDs. Following the fabrication of the micro-LEDs, we demonstrated that the SQW structure of InGaN-based red micro-LEDs resulted in higher EQE, maximum bandwidth, and faster transmission rates in non-return-to-zero on–off keying (NRZ-OOK) modulation format. The SQW structure of InGaN-based red micro-LEDs serves as a foundation for future developments in micro-LED technology and holds significant potential to revolutionize the next-generation full-color display and VLC industries.

## Materials

**Fig. 1:**
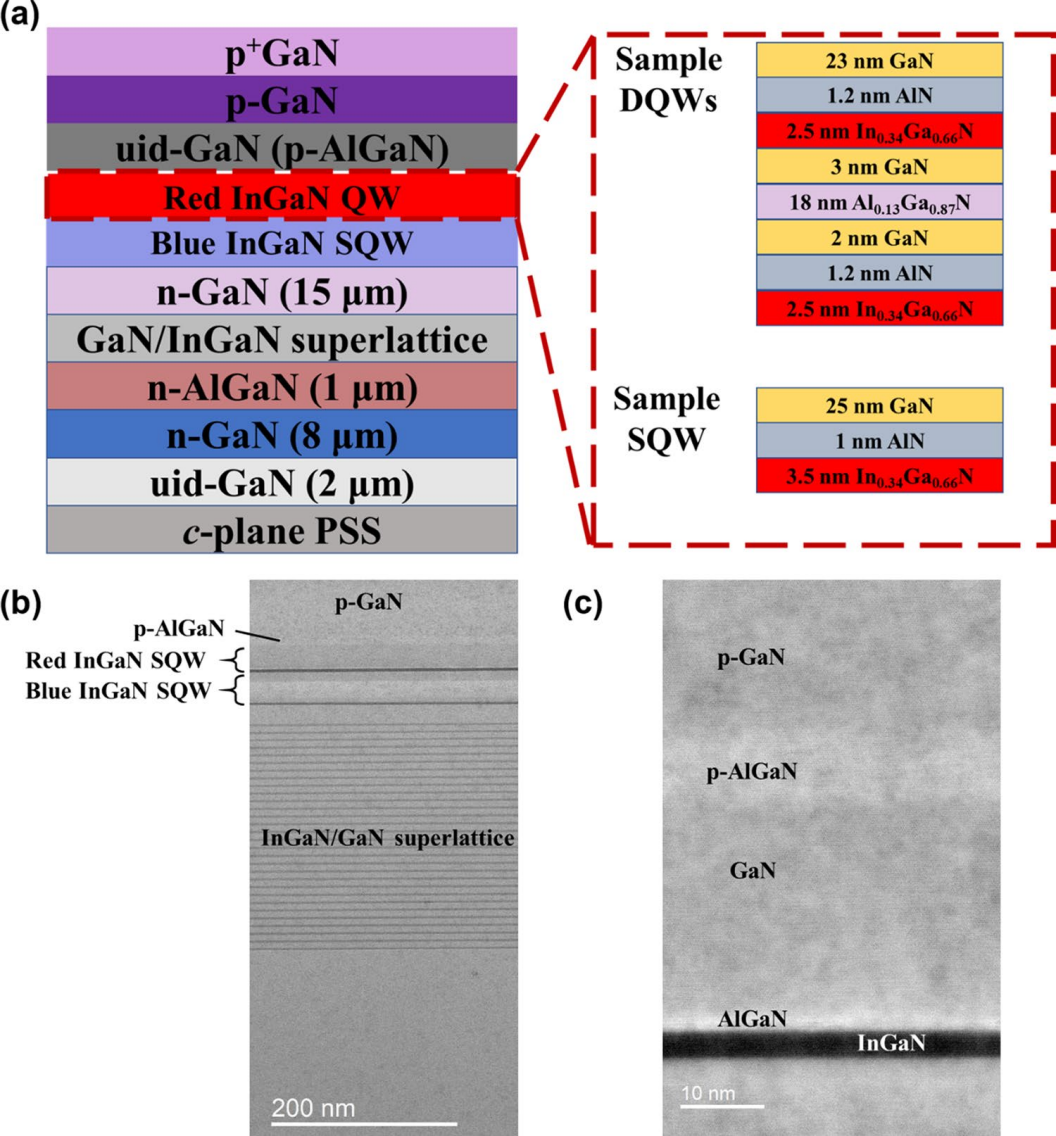
**a** Schematic illustration of the InGaN-based red LED structures for samples DQWs and SQW. **b** Cross-sectional TEM image of the sample SQW. **c** Zoom-in cross-sectional TEM image around the QW region of the SQW sample

Figure 1a displays a diagram of two InGaN red epitaxial with varying QW designs. The InGaN-based LED structures were grown on a conventional *c*-plane patterned sapphire substrate (PPS) via metal–organic vapor-phase epitaxy (MOVPE). The epitaxial process started with a 2 μm unintentionally doped (uid) GaN layer, followed by an 8 μm n-GaN, 1 μm Si-doped n-AlGaN layers, pairs of GaN/In_0.08_Ga_0.92_N superlattice (SL) layers and 15 μm n-GaN layer. Subsequently, a low-In-content blue InGaN SQW and high-In-content red QW were grown as the active layer. The main difference between the two samples is the number of the red InGaN QW layer: double (sample DQWs) and single (sample SQW). The insets in Fig. 1 depict the detailed structure of the red InGaN QWs. In sample DQWs, the QW is composed of In_0.34_Ga_0.66_N DQWs and AlN/GaN/Al_0.13_Ga_0.87_N/GaN barrier layers, with GaN replacing the Al_0.13_Ga_0.87_N part in the upper barrier. In contrast, sample SQW has an In_0.34_Ga_0.66_N SQW with AlN/GaN barrier layers. Finally, uid-GaN or p-AlGaN, Mg-doped p-GaN, and heavily Mg-doped p^+^-GaN contact layers were grown [17, 29]. The TEM images of sample SQW are shown in Fig. 1b, c. By reducing the numbers of the high-In-content QW, the compositional uniformity of the materials and better quality in the active region are presented via cross-sectional images [17]. Furthermore, it is expected that the strain accumulation on the sample will be mitigated.

## Results and discussion

### Materials quality

**Fig. 2:**
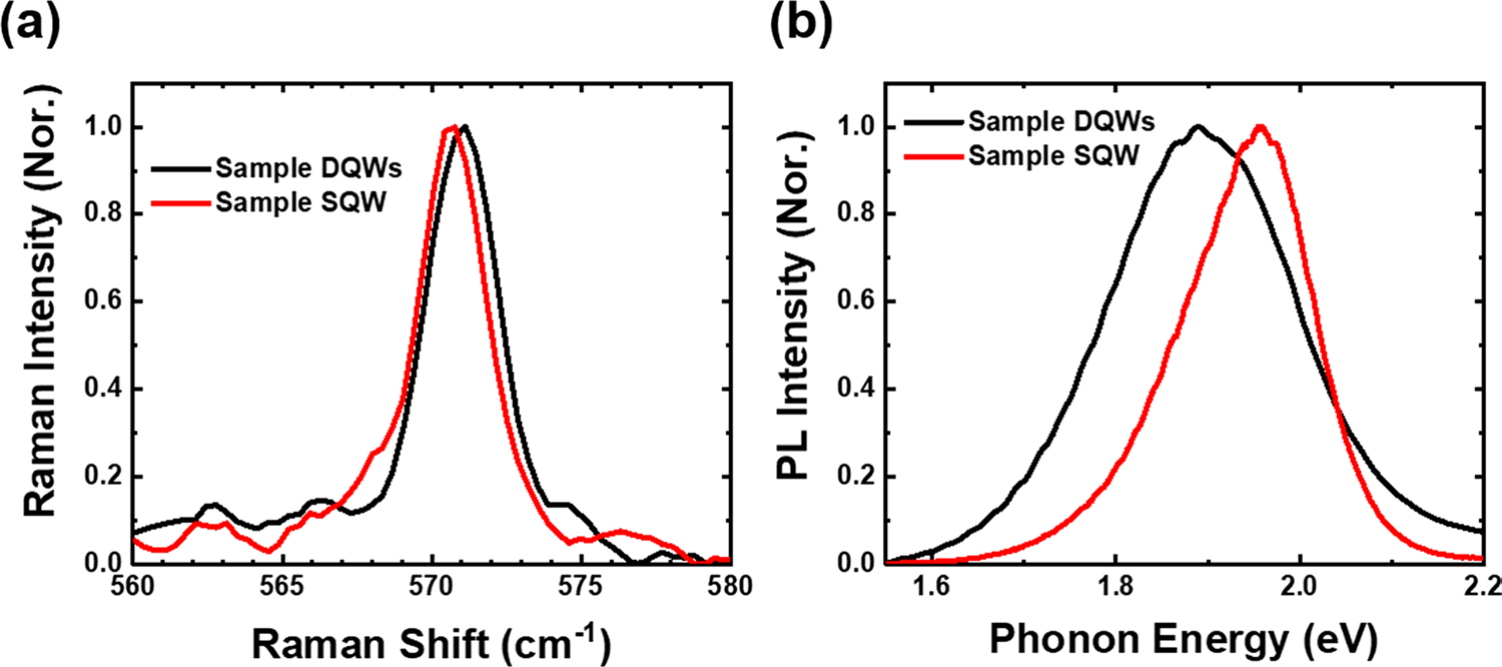
Spectra comparison between samples DQWs and SQW. **a** Measured Raman spectra of the E_2_ mode of samples DQWs and SQW. Slight frequency shifts and narrower FWHM can be observed for sample SQW. **b** PL spectra at room temperature for both samples. Sample SQW is narrower as well

To investigate the optical, structural, and electrical characteristics of InGaN/GaN materials, powerful instruments such as Raman and photoluminescence (PL) measurements have proven to be effective. Raman signals from the samples are discriminated by the MS 5204i monochromator (SOL instruments) and detected by the DR-324B-FI-601 CCD detector (Oxford Instruments). PL spectra are measured by the iHR550 spectrometer (HORIBA). In particular, Raman spectroscopy was used to measure the high-energy Raman E_2_ mode to assess the stress in InGaN-based red micro-LEDs. The laser spot was focused on the surface of the epitaxial structure of p^+^-GaN, and the accumulated strain of the QWs could be detected via Raman spectroscopy. Figure 2a illustrates the normalized (Nor.) Raman spectra of E_2_ mode for samples DQWs and SQW at room temperature. The peak position of the E_2_ mode is mainly influenced by strain and can be used as a metric for the strain state of a layer. In Fig. 2a, the peaks of the E_2_ mode for samples DQWs and SQW are at 571.01 and 570.66 cm^−1^, respectively. Typically, the formula below can be used to compute the stress in the GaN layers:

1 $$\sigma \left( {{\text{Raman}}} \right) = \frac{\Delta \omega }{k}$$

where $\sigma$ represents the stress value of the epitaxial layer, $\Delta \omega$ is the frequency difference between the high-energy *E*_2_ mode peak of GaN and the stress-free GaN crystal value of 567.36 cm^−1^, and $k$ denotes the stress coefficient of approximately 2.56 cm^−1^ GPa^−1^ [32, 33]. Both the sample DQWs and SQW exhibit a redshift in the E_2_ peak compared to stress-free GaN, suggesting the existence of compressive strain in the InGaN-based red micro-LEDs. The larger Raman shift of sample DQWs suggests a more significant compressive strain than that of sample SQW. Moreover, based on $\Delta \omega$, the compressive stress values of samples DQWs and SQW were calculated to be 1.43 GPa and 1.29 GPa, respectively. The lower biaxial stress value of sample SQW indicates that its structure is subject to less strain. Additionally, the full width at half maximum (FWHM) of the peak can serve as another indication of defect inclusion in the layer. Strain gradients or phonon-defect scattering may result in broadening the peak. As shown in Fig. 2a, the FWHM of the E_2_ Raman spectrum for samples DQWs and SQW are 2.97 and 2.74 cm^−1^, respectively. With a slightly narrower FWHM, sample SQW is believed to alleviate strain accumulation on the surface, suppress defect generation, and possess better crystal quality in the QW active layers.

The normalized photoluminescence (PL) spectra for both samples at room temperature are presented in Fig. 2b. The peak energy of ~ 1.9 eV suggests that the emission originates from the high-In-content QWs. The FWHM of the PL spectra for samples DQWs and SQW is 0.239 and 0.167 eV, respectively. Generally, the large lattice mismatch between InN and GaN and their low miscibility lead to the aggregation of indium, which can cause nonuniform distribution of indium due to the accumulation of strain. This nonuniform distribution of indium can create spatial potential fluctuations for trapping carriers, resulting in carriers transporting among different levels of states and broadening the spectra [34, 35]. The existence of defect states between the conduction band and valance band can also lead to broadening the PL spectra linewidth. The broader FWHM of 0.239 eV in sample DQWs suggests that there are more potential fluctuations due to the relatively nonuniform distribution of indium. Therefore, the material quality of sample DQWs is relatively inferior to sample SQW, which is consistent with the Raman spectra. The broader FWHM of sample DQWs could also indicate the existence of multiple additional emissions, which degrades the color purity. The Raman and PL spectra demonstrate less strain accumulation and a narrower FWHM in the sample SQW. Hence, substituting the DQWs structure with SQW could improve the crystalline quality, uniformity of indium distribution, and emission purity of the InGaN-based active layer.

### Temperature-dependent PL and time-resolved PL

**Fig. 3:**
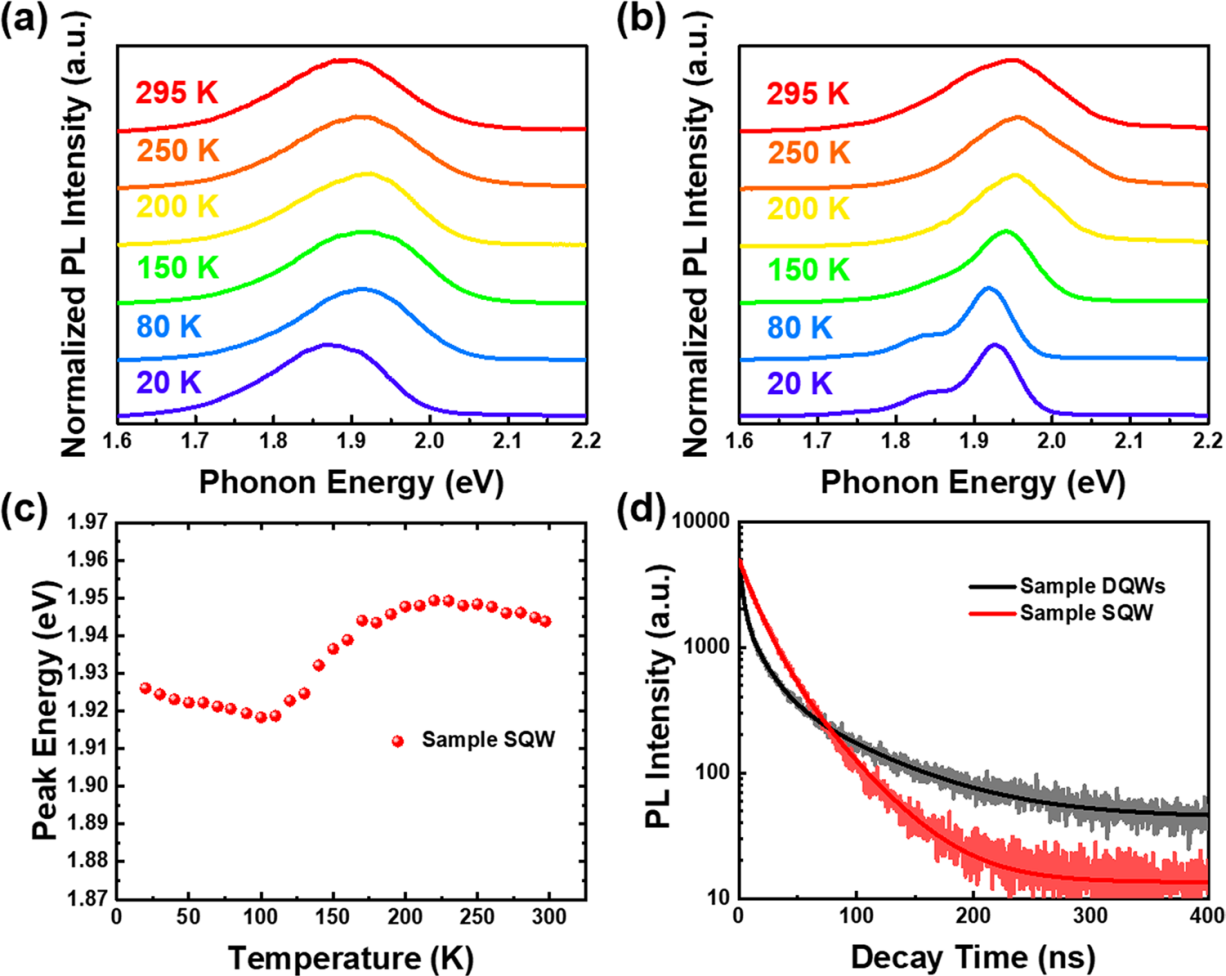
Temperature-dependent PL spectra. PL spectra at various temperatures of **a** sample DQWs and **b** sample SQW. **c** Temperature-dependent PL peak energy of sample SQW. The peak shift in S-shape is shown. **d** TRPL curves of samples DQWs and SQW at room temperature

In this section, we analyzed the optical properties of the two samples by measuring their PL in a temperature range from 20 to 295 K. Figure 3a, b shows the normalized PL spectra of samples DQWs and SQW, respectively. The PL FWHM of sample SQW is consistently narrower than that of sample DQWs at all temperatures, which aligns with the results from the previous section and confirms the presence of more potential fluctuations in sample DQWs. Moreover, PL peak shifts were observed for both samples as the temperature increased; nevertheless, the shifts exhibit distinct behaviors. In sample DQWs, the PL peak first blueshifts, then redshifts with increasing temperature. The broader FWHM could be the evidence of a more nonuniform distribution of indium in space, indicating that indium-rich clusters or even quantum dot (QD)-like localization centers might exist in the active layers of sample DQWs [36–38]. These clusters and QD-like localization centers may cause structural geometric constraints, leading to a high binding energy of InGaN QD-like exciton. Thus, we suggest that strong excitonic effects dominate in sample DQWs. Due to the strong binding energy, excitons are prevented from dissociation and delocalization at low temperatures. Instead, the excitons are confined and accumulated in the localized states, occupying the higher energy states as temperature increases, resulting in a PL peak blueshift. This indicates that strong localization exists in the DQWs sample at low temperatures [39]. On the contrary, the excitons are thermally activated and dissociated at higher temperatures, causing free carriers to randomly distribute in the localized states. Besides, as predicted by the well-known Varshni equation, temperature-induced bandgap shrinkage begins to predominate in the high-temperature range, leading to a decrease in PL peak energy [40].

On the other hand, as depicted in Fig. 3b, c, sample SQW shows an S-shaped temperature-dependent luminescence peak energy behavior, which is commonly noticed in InGaN QWs [41–43]. In general, this S-shaped temperature-dependent curve is considered a sign of the presence of localization states. The underlying explanation for the S-shaped temperature-dependent behavior is as follows [44]: at extremely low temperatures, the injected carriers are distributed haphazardly across all localized states in QWs. Since the mobility of the photo-generated carriers is too low at this stage, they cannot reach the deep localization states, and therefore, they undergo recombination and emit photons across all localized states. As temperature increases, the PL peak shifts toward the red end of the spectrum because more carriers can now migrate into the deep localized states and recombine from the low energy levels. Moreover, with the increase in temperature, a greater number of carriers are then thermally excited out of the deep localized states and into the shallower ones with higher energy levels, resulting in a blue shift of the emission peak. At even higher temperatures, due to the heightened thermal energy, most carriers are not trapped in the deep localized states and are instead randomly redistributed across all localized states. Consequently, the peak energy decreases owing to the temperature-induced bandgap shrinkage [40].

We conducted time-resolved PL (TRPL) measurements for both samples to further investigate their properties. Figure 3d shows the TRPL spectra of both samples at room temperature. The decay curves were fitted using a multi-exponential function as follows:

2 $$I\left( t \right) = \mathop \sum \limits_{i} A_{i} \times \exp \left( {\frac{ - t}{{\tau_{i} }}} \right)$$

where $I\left( t \right)$ is the TRPL intensity at time $t$, $\tau_{i}$ represents the lifetime, and $A_{i}$ represents the corresponding pre-exponential functions. We used three and two exponential decay models to evaluate PL lifetimes for sample DQWs and SQW, respectively, and the fitted lifetime parameters are presented in Table 1. In sample DQWs, owing to the presence of QD-like localization centers and excitonic effects, a fast decay with the shortest lifetime $\tau_{1}$, only sub nanoseconds, can be attributed to trion lifetime [45, 46]. In addition, non-radiative recombination tends to dominate at room temperature, and the ratio of $A_{2}$ is larger than $A_{3}$. Thus, the short and long decay lifetimes $\tau_{2}$ and $\tau_{3}$ for both samples are attributed to non-radiative recombination and radiative recombination lifetimes, respectively. It is found that the time constants of non-radiative lifetime have similar values. However, the $\tau_{3}$ value, which represents the radiative recombination lifetime, shows a significant difference between the two samples. The $\tau_{3}$ value of 39.18 ns for sample SQW is noticeably shorter than that for sample DQWs (73.19 ns). Furthermore, the average lifetime $\tau_{{{\text{avg}}}}$ is calculated using the following equation:

3 $$\tau_{{{\text{avg}}}} = \mathop \sum \limits_{i} \frac{{A_{i} \tau_{i}^{2} }}{{A_{i} \tau_{i} }}$$

**Table 1:** Fitting parameters of two samples

Parameters	*A*_1_ (%)	$\tau_{1}$(ns)	*A*_2_ (%)	$\tau_{2}$(ns)	*A*_3_ (%)	$\tau_{3}$(ns)
Sample DQWs	61.66	3.06	28.58	15.63	9.76	73.19
Sample SQW	–	–	71.65	14.76	28.35	39.18

According to Eq. (3), the $\tau_{{{\text{avg}}}}$ values of sample DQWs and SQW are approximately 44.34 and 27.27 ns, respectively. The results indicate that the carriers have a faster transition rate during emission for sample SQW.

Additionally, we can use the carrier lifetimes to estimate the IQE of the samples. We defined the recombination rate *k* as $k = 1/\tau$. As only radiative recombination contributes to PL intensity, the relative PL efficiency reflects the ratio of the radiative recombination rate to the total recombination rate:

4 $$\eta = \frac{{k_{{\text{r}}} }}{{k_{{\text{r}}} + k_{{{\text{nr}}}} }}$$

where $\eta$ denotes the IQE of the samples, $k_{{\text{r}}}$ stands for radiative recombination rate, and $k_{{{\text{nr}}}}$ represents non-radiative recombination rate. The IQE of samples DQWs and SQW was found to be 17.60% and 27.36%, respectively. It is known that there is a positive correlation between IQE and EQE. Therefore, we can expect an improvement in EQE since the IQE of the sample SQW is 55.5% higher than that of sample DQWs

### EL spectra and electrical properties

**Fig. 4:**
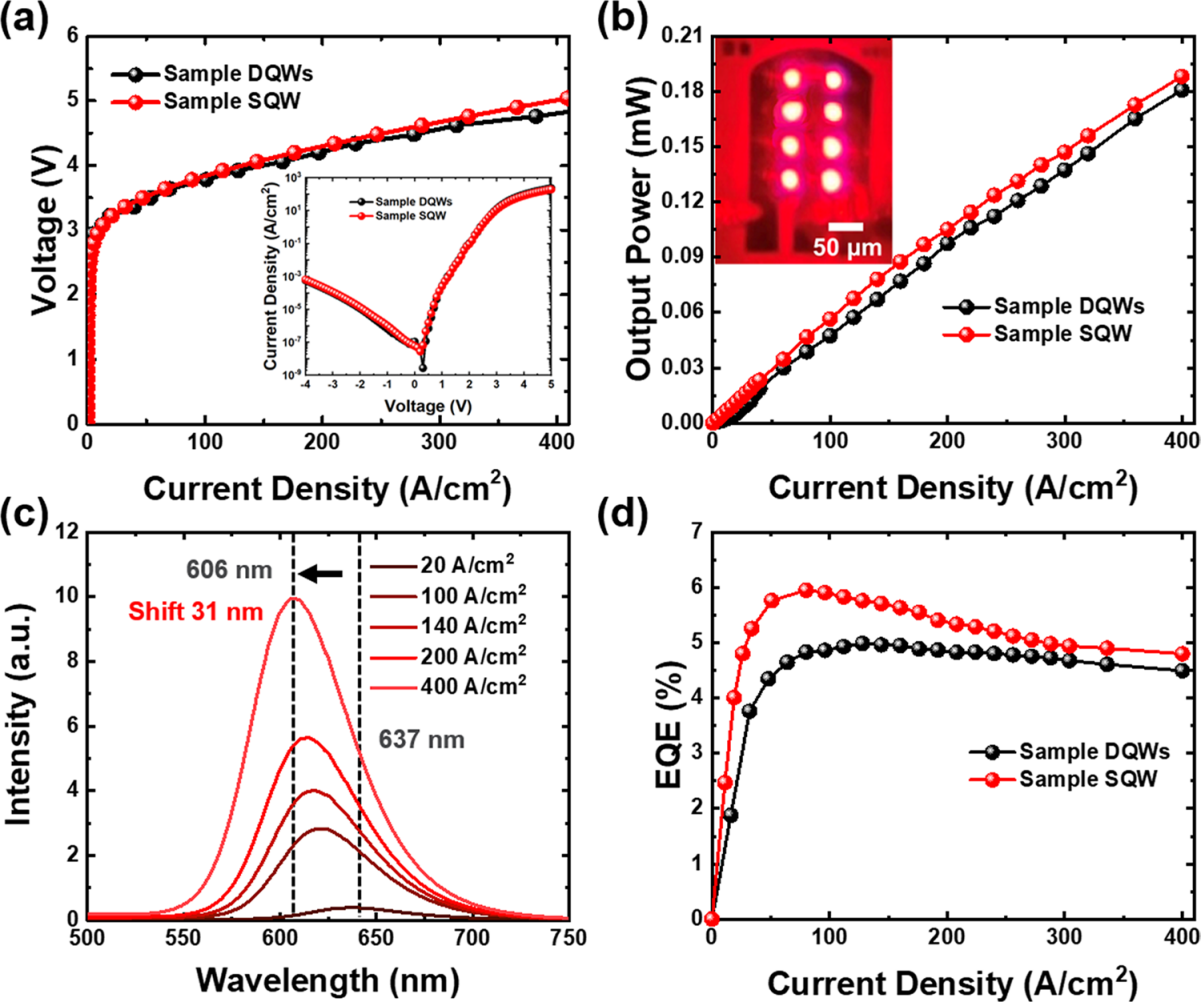
Device performance. **a**, **b** Voltage and output power as a function of current density. The inset in **b** shows the illumination image of sample SQW. **c** EL spectra of sample SQW at different current densities. **d** EQE-current density curves of micro-LEDs

After analyzing the structural and optical performance of the epitaxial crystal structure, the samples were subjected to the micro-LEDs fabrication process. Circular mesas with a diameter of 25 μm were used to make micro-LEDs based on samples DQWs and SQW, respectively. The use of the atomic layer deposition (ALD) technique helped to passivate the etched mesas and reduce the leakage currents of the devices. Additionally, the introduction of distributed Bragg reflector (DBR) at the bottom of the devices improved the efficiency of light extraction. The detailed micro-LED process flow can be found in our previous work [29]. The device performance of samples DQWs and SQW is summarized in Fig. 4. The forward voltage and output power as a function of the injection current density of devices are shown in Fig. 4a, b, respectively. The inset in Fig. 4b shows the fabricated 25 μm × 8 red-emitting micro-LED array based on sample SQW. The current density is normalized by the active region area. As shown in Fig. 4a, b, the light–current density–voltage (*L*–*J*–*V*) characteristics for both samples exhibit similar performance, indicating that there is no significant difference between the two devices. However, it is worth noting that the forward voltage of sample SQW is slightly lower at 3.22 V at 20.62 A/cm^2^. While conducting a reverse voltage of − 4 V, the reverse leakage current density reaches around 10^–3^ A/cm^2^. The relation between current density and output power is almost linear before the output power reaches 0.2 mW for both samples, while the output power is kind of larger for sample SQW in all ranges of current density. The results suggest that, despite the difference in the number of QW layers, the emission optical properties of the SQW active layer structure are comparable to those of the DQWs structure.

The electroluminescence (EL) emission spectra of the SQW device are presented in Fig. 4c. The initial wavelength, starting at 637 nm with 20 A/cm^2^ injection current density, shifts toward 606 nm as the current density is increased to 400 A/cm^2^. This blueshift phenomenon in InGaN-based micro-LEDs is caused by the screening effect of the polarization field and the band-filling effect in the localized energy states of the InGaN QW, which becomes dominant at higher injection current densities. In comparison with our previous work, a longer emission wavelength of 652 nm was observed at low current density for the DQWs device [29]; however, a much larger blueshift of 38 nm occurred as the current density increased. Moreover, the FWHM at 400 A/cm^2^ of the SQW device is 61.2 nm, which is narrower than that of the DQWs device. Both the blueshift and FWHM behavior imply that the SQW structure can achieve a higher purity of red emission.

Figure 4d demonstrates the EQE values as a function of injection current density. The maximum EQE values achieved are 4.99% and 5.95% for DQWs and SQW devices, respectively. This signifies that the EQE of the SQW device is improved by nearly 20% compared to that of the DQWs device. The EQE value is considered high for red emission with an emission peak wavelength over 620 nm [14, 15, 29, 47, 48]. The maximum EQE value for the SQW device is achieved at a slightly lower current density of 80 A/cm^2^. Even though the sidewalls have been passivated via ALD techniques, the efficiency droop phenomenon is still inevitable in a high current density state. The EQE of the sample SQW drops from 5.95 to 4.80% with a droop efficiency of 19.33% due to the QCSE in the polar *c*-plane GaN. However, it is worth noting that the EQE values for sample SQW micro-LED are consistently higher than those of the DQWs micro-LED until the current density reaches 400 A/cm^2^. The outstanding electrical properties of the SQW structure suggest its potential in realizing highly efficient red micro-LEDs.

### Modulation bandwidth and transmission data rate

The modulation bandwidth of the LED is one of the critical parameters in VLC applications. To measure the frequency response and access the VLC performance of the micro-LEDs, we utilized a vector network analyzer (VNA, HP8720ES). A bias tee was used to link the VNA-generated alternating current signal to a direct current. Then, we delivered the coupled signal into micro-LEDs using a microscope (ACP40-GS-250), which produced an optical signal with a small signal. After that, we used a plastic optical fiber to collect the optical signals from the micro-LEDs and transport them to a photodetector (Graviton, SPA-3). The photodetector would convert the optical signals into electrical signals and analyze the frequency response via VNA. Generally, the modulation bandwidth of LEDs is affected by RC time constants and recombination lifetimes. Nevertheless, in the case of LEDs with dimensions of 100 μm × 100 μm or less, the modulation bandwidth is primarily governed by the radiative lifetime. This is attributed to the small geometric capacitance, which effectively prevents the RC time constants from taking precedence [49]. Therefore, we can describe the − 3 dB bandwidth of the micro-LEDs using the following equation:

5 $$f_{{ - 3{\text{ dB}}}} = \frac{\sqrt 3 }{{2\pi \tau }}$$

where $f_{{ - 3{\text{ dB}}}}$ and $\tau$ represent electrical − 3 dB bandwidth and the carrier lifetime, respectively. The recombination lifetimes are usually clarified using the ABC rate equation model, which is expressed as:

6 $$I = e \cdot a \cdot d\left( {An + Bn^{2} + Cn^{3} } \right)$$

where $n$ is the carrier density in the active region, $A$, $B$, and $C$ are the Shockley–Read–Hall (SRH), radiative, and Auger coefficients, respectively. The electronic charge, active region area, and thickness are presented by $e$, $a$, and $d$, respectively. The carrier lifetime can be determined by the recombination rate to the carrier density, given as follows:

7 $$\tau^{ - 1} = A + 2Bn + 3Cn^{2}$$

**Fig. 5:**
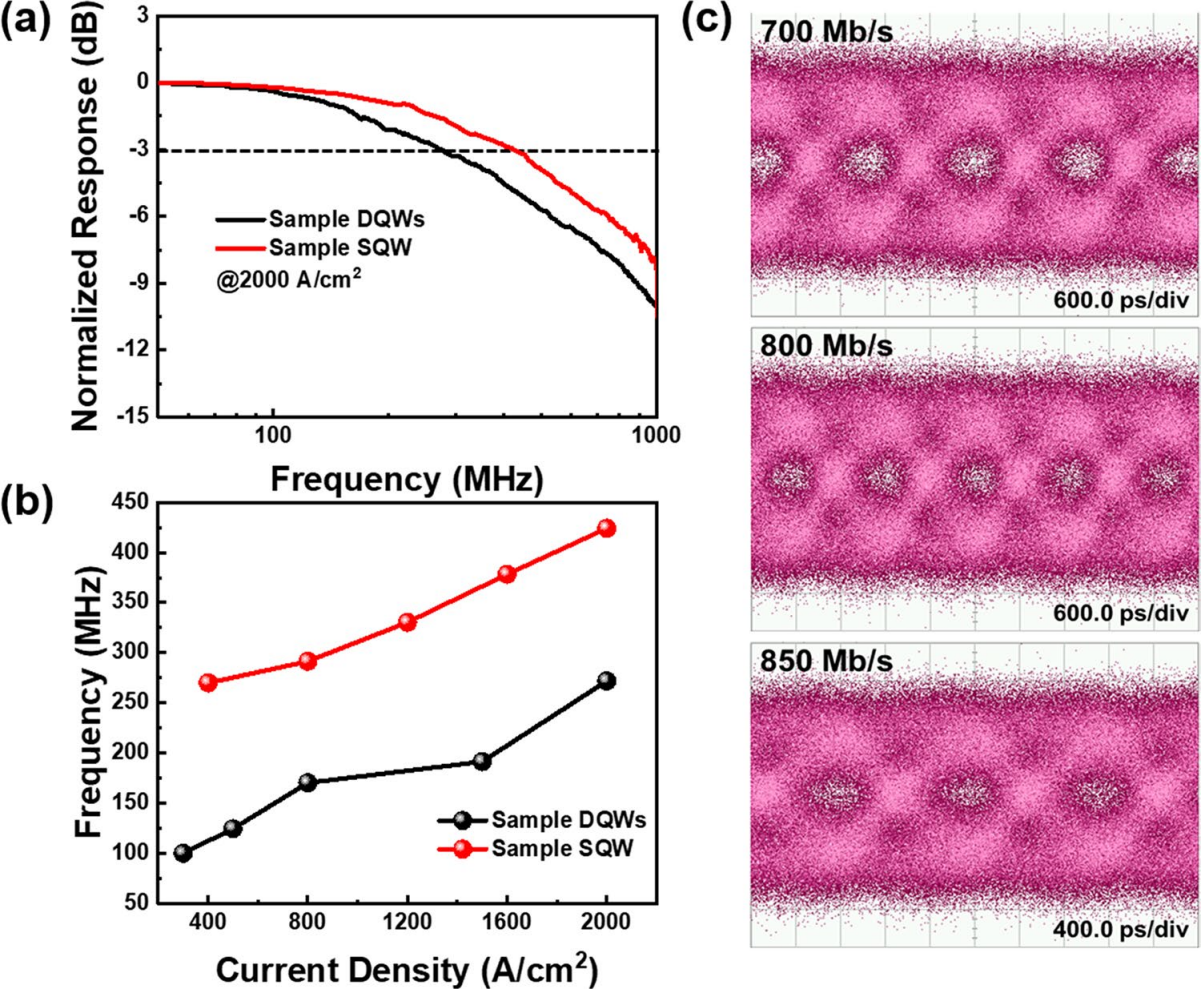
**a** Normalized frequency response for samples DQWs and SQW at 2000 A/cm^2^. **b** The frequency as a function of current density for samples DQWs and SQW. **c** NRZ-OOK eye diagrams for sample SQW at 700 Mbit/s, 800 Mbit/s, and 850 Mbit/s

In Fig. 5a, we can see the corresponding frequency response for samples DQWs and SQW at 2000 A/cm^2^. It is noted that the emission wavelength shifts to the yellow range for both samples under such high current injection. The micro-LEDs exhibited a maximum − 3 dB bandwidth of 424 MHz for sample SQW at 2000 A/cm^2^, which is higher than the results for sample DQWs and our previous work [29]. According to Eq. (5), this higher bandwidth can also be inferred from the faster decay rate for SQW epitaxial structure in the TRPL measurement. As claimed by Eqs. (5), (6), and (7), the equations demonstrate that an increase in injection current leads to a reduction in the differential carrier lifetime, resulting in an improvement in the − 3 dB bandwidth. Therefore, the − 3 dB bandwidth is directly proportional to the current density, and the maximum achievable bandwidth can be obtained at high current densities, as shown in Fig. 5b. These observations indicate that the RC delay does not limit the − 3 dB bandwidth under the given operating conditions. Consequently, it can be inferred that reducing the carrier lifetime can lead to higher modulation bandwidths [50, 51].

As sample SQW achieved a higher − 3 dB bandwidth, it is worth investigating its transmission data rate. The transmission performance of the micro-LED was demonstrated on an off–on keying (OOK) system using a non-return-to-zero (NRZ) 2^7^–1 pseudorandom binary sequence (PRBS7) generated by a bit pattern generator (Anritsu MP1800A). The eye diagrams were analyzed and recorded by an 86100A oscilloscope, and the detected NRZ-OOK eye diagrams at 700 Mbit/s, 800 Mbit/s, and 850 Mbit/s are shown in Fig. 5c. The eye is not very clear but still opened at 700 Mbit/s and gradually becomes smaller. It is not until 850 Mbit/s that the eye area is almost closed because of the comparatively low signal-to-noise ratio (SNR). In addition, the bit error rate (BER) of 800 Mbit/s is 2.6 × 10^–3^, satisfying the forward error correction (FEC) threshold limit of 3.8 × 10^–3^. This indicates that the micro-LEDs have the potential for application in data rates around 800 Mbit/s. The measured bandwidth and data transmission rates are higher than those for sample DQWs, suggesting that InGaN red micro-LEDs with SQW structure hold great promise for high-speed VLC applications.

## Conclusion

In summary, our study showcases the exceptional performance of InGaN-based red micro-LEDs with SQW structure during epitaxial growth. By comparing the results with the InGaN red-LED with DQWs structure, the reduction of the number of QW layers in the SQW structure reduces strain, resulting in a uniform distribution of indium and higher internal IQE, due to improved crystal quality. The superiority of the SQW structure is evident from the narrower FWHM of the emission spectrum, reducing from 0.239 to 0.167 eV, which results in higher emission purity, as confirmed through Raman, PL, and TRPL measurements. Additionally, the use of a 25-μm-sized (the diameter of circular mesas) micro-LED with the SQW structure exhibits stability, as evidenced by a smaller blueshift (31 nm) with increasing injection current density from 20 to 400 A/cm^2^, and demonstrates a higher maximum EQE of approximately 5.95%. At high injection densities, the device achieves a maximum − 3 dB bandwidth of 424 MHz, with data rates reaching up to 800 Mbit/s using the NRZ-OOK modulation format. Further reduction in the carrier lifetime is suggested to yield higher modulation bandwidths. These results demonstrate that InGaN-based red micro-LEDs with the SQW structure not only maintain high efficiency but also enhance data transmission rates, holding considerable potential for future applications in high-speed VLC and full-color micro-display systems.

## Data Availability

The data presented in this study are available from the corresponding author upon reasonable request.

## References

[1] Lee TY, Chen LY, Lo YY, Swayamprabha SS, Kumar A, Huang YM, Chen SC, Zan HW, Chen FC, Horng RH, Kuo HC (2022). Technology and applications of micro-LEDs: their characteristics, fabrication, advancement, and challenges. ACS Photonics.

[2] Smith JM, Ley R, Wong MS, Baek YH, Kang JH, Kim CH, Gordon MJ, Nakamura S, Speck JS, DenBaars SP (2020). Comparison of size-dependent characteristics of blue and green InGaN microLEDs down to 1 μm in diameter. Appl Phys Lett.

[3] Hwang D, Mughal A, Pynn CD, Nakamura S, DenBaars SP (2017). Sustained high external quantum efficiency in ultrasmall blue III–nitride micro-LEDs. Appl Phys Express.

[4] Fan K, Tao J, Zhao Y, Li P, Sun W, Zhu L, Lv J, Qin Y, Wang Q, Liang J, Wang W (2022). Size effects of AlGaInP red vertical micro-LEDs on silicon substrate. Results Phys.

[5] Huang HH, Huang SK, Tsai YL, Wang SW, Lee YY, Weng SY, Kuo HC, Lin CC (2020). Investigation on reliability of red micro-light emitting diodes with atomic layer deposition passivation layers. Opt Express.

[6] Li YY, Lin FZ, Chi KL, Weng SY, Lee GY, Kuo HC, Lin CC (2021). Analysis of size-dependent quantum efficiency in AlGaInP micro-light-emitting diodes with consideration for current leakage. IEEE Photon J.

[7] Zhang S, Zhang J, Gao J, Wang X, Zheng C, Zhang M, Wu X, Xu L, Ding J, Quan Z, Jiang F (2020). Efficient emission of InGaN-based light-emitting diodes: toward orange and red. Photonics Res.

[8] Zhuang Z, Iida D, Ohkawa K (2021). Ultrasmall and ultradense InGaN-based RGB monochromatic micro-light-emitting diode arrays by pixilation of conductive p-GaN. Photonics Res.

[9] Takeuchi T, Sota S, Katsuragawa M, Komori M, Takeuchi H, Amano H, Akasaki I (1997). Quantum-confined Stark effect due to piezoelectric fields in GaInN strained quantum wells. Jpn J Appl Phys.

[10] Langer T, Kruse A, Ketzer FA, Schwiegel A, Hoffmann L, Jönen H, Bremers H, Rossow U, Hangleiter A (2011). Origin of the “green gap”: increasing nonradiative recombination in indium-rich GaInN/GaN quantum well structures. Phys Status Solidi C.

[11] Vaitkevičius A, Mickevičius J, Dobrovolskas D, Tuna Ö, Giesen C, Heuken M, Tamulaitis G (2014). Influence of quantum-confined Stark effect on optical properties within trench defects in InGaN quantum wells with different indium content. J Appl Phys.

[12] Tawfik WZ, Hyeon GY, Lee JK (2014). Stress-induced piezoelectric field in GaN-based 450-nm light-emitting diodes. J Appl Phys.

[13] Tawfik WZ, Hyun GY, Ryu SW, Ha JS, Lee JK (2016). Piezoelectric field in highly stressed GaN-based LED on Si(111) substrate. Opt Mater.

[14] Pasayat SS, Gupta C, Wong MS, Ley R, Gordon MJ, DenBaars SP, Nakamura S, Keller S, Mishra U (2021). Demonstration of ultra-small (< 10 μm) 632 nm red InGaN micro-LEDs with useful on-wafer external quantum efficiency (> 0.2%) for mini-displays. Appl Phys Express.

[15] Iida D, Zhuang Z, Kirilenko P, Velazquez-Rizo M, Najmi MA, Ohkawa K (2020). 633-nm InGaN-based red LEDs grown on thick underlying GaN layers with reduced in-plane residual stress. Appl Phys Lett.

[16] Chan P, Rienzi V, Lim N, Chang HM, Gordon M, DenBaars SP, Nakamura S (2021). Demonstration of relaxed InGaN-based red LEDs grown with high active region temperature. Appl Phys Lett.

[17] Iida D, Kirilenko P, Velazquez-Rizo M, Zhuang Z, Najmi MA, Ohkawa K (2022). Demonstration of 621-nm-wavelength InGaN-based single-quantum-well LEDs with an external quantum efficiency of 4.3% at 10.1 A/cm^2^. AIP Adv.

[18] Pandey A, Xiao Y, Reddeppa M, Malhotra Y, Liu J, Min J, Wu Y, Mi Z (2023). A red-emitting micrometer scale LED with external quantum efficiency> 8%. Appl Phys Lett.

[19] Sadat H, Abaza M, Mansour A, Alfalou A (2022). A survey of NOMA for VLC systems: research challenges and future trends. Sensors.

[20] Abaza M, Mesleh R, Mansour A, Aggoune EH (2015). Performance analysis of MISO multi-hop FSO links over log-normal channels with fog and beam divergence attenuations. Opt Commun.

[21] Kavehrad M (2007). Broadband room service by light. Sci Am.

[22] Singh KJ, Huang WT, Hsiao FH, Miao WC, Lee TY, Pai YH, Kuo HC (2023). Recent advances in micro-LEDs having yellow-green to red emission wavelengths for visible light communications. Micromachines.

[23] Khalid AM, Cossu G, Corsini R, Choudhury P, Ciaramella E (2012). 1-Gb/s transmission over a phosphorescent white LED by using rate-adaptive discrete multitone modulation. IEEE Photonics J.

[24] Singh KJ, Huang YM, Ahmed T, Liu AC, Huang Chen SW, Liou FJ, Wu T, Lin CC, Chow CW, Lin GR, Kuo HC (2020). Micro-LED as a promising candidate for high-speed visible light communication. Appl Sci.

[25] Huang Y, Guo Z, Wang X, Li H, Xiang D (2020). GaN-based high-response frequency and high-optical power matrix micro-LED for visible light communication. IEEE Electron Device Lett.

[26] Lin GR, Kuo HC, Cheng CH, Wu YC, Huang YM, Liou FJ, Lee YC (2021). Ultrafast 2 × 2 green micro-LED array for optical wireless communication beyond 5 Gbit/s. Photonics Res.

[27] Xu F, Jin Z, Tao T, Tian P, Wang G, Liu X, Zhi T, Yan Q, Pan D, Xie Z, Xu K, Liu B, Zhang R (2022). C-plane blue micro-LED with 1.53 GHz bandwidth for high-speed visible light communication. IEEE Electron Device Lett.

[28] Xu F, Qiu P, Tao T, Tian P, Liu X, Zhi T, Xie Z, Liu B, Zhang R (2023). High bandwidth semi-polar InGaN/GaN micro-LEDs with low current injection for visible light communication. IEEE Photonics J.

[29] Huang YM, Peng CY, Miao WC, Chiang H, Lee TY, Chang YH, Singh KJ, Iida D, Horng RH, Chow CW, Lin CC, Ohkawa K, Chen SC, Kuo HC (2022). High-efficiency InGaN red micro-LEDs for visible light communication. Photonics Res.

[30] Lan HY, Tseng IC, Kao HY, Lin YH, Lin GR, Wu CH (2018). 752-MHz modulation bandwidth of high-speed blue micro light-emitting diodes. IEEE J Quantum Electron.

[31] Cai S, Wang D, Zeng N, Li K, Wu Q, Yin Y (2021). Influence of multiple quantum well number on modulation bandwidth of InGaN/GaN light-emitting diodes. J Opt.

[32] Zhou S, Liu X, Yan H, Gao Y, Xu H, Zhao J, Quan Z, Gui C, Liu S (2018). The effect of nanometre-scale V-pits on electronic and optical properties and efficiency droop of GaN-based green light-emitting diodes. Sci Rep.

[33] Zeng Y, Ning J, Zhang J, Jia Y, Yan C, Wang B, Wang D (2020). Raman analysis of E_2_ (high) and A_1_ (LO) phonon to the stress-free GaN grown on sputtered AlN/graphene buffer layer. Appl Sci.

[34] Lin YS, Ma KJ, Hsu C, Feng SW, Cheng YC, Liao CC, Yang CC, Chou CC, Lee CM, Chyi JI (2000). Dependence of composition fluctuation on indium content in InGaN/GaN multiple quantum wells. Appl Phys Lett.

[35] Feng SW, Cheng YC, Chung YY, Yang CC, Mao MH, Lin YS, Ma KJ, Chyi JI (2002). Multiple-component photoluminescence decay caused by carrier transport in InGaN/GaN multiple quantum wells with indium aggregation structures. Appl Phys Lett.

[36] Amloy S, Karlsson KF, Eriksson MO, Palisaitis J, Persson POÅ, Chen YT, Chen KH, Hsu HC, Hsiao CL, Chen LC (2014). Excitons and biexcitons in InGaN quantum dot like localization centers. Nanotechnology.

[37] Meng Y, Wang L, Zhao G, Li F, Li H, Yang S, Wang Z (2018). Red emission of InGaN/GaN multiple-quantum-well light-emitting diode structures with indium-rich clusters. Phys Status Solidi A.

[38] Cheong MG, Liu C, Choi HW, Lee BK, Suh EK, Lee HJ (2003). Study of the origin of luminescence in high indium composition InGaN/GaN quantum wells. J Appl Phys.

[39] Hsiao FH, Miao WC, Hong YH, Chiang H, Ho IH, Liang KB, Iida D, Lin CL, Ahn H, Ohkawa K, Chang CY, Kuo HC (2023). Structural and optical analyses for InGaN-based red micro-LED. Discover Nano.

[40] Varshni YP (1967). Temperature dependence of the energy gap in semiconductors. Physica.

[41] Cho YH, Gainer GH, Fischer AJ, Song JJ, Keller S, Mishra UK, DenBaars SP (1998). “S-shaped” temperature-dependent emission shift and carrier dynamics in InGaN/GaN multiple quantum wells. Appl Phys Lett.

[42] Chowdhury AM, Roul B, Singh DK, Pant R, Nanda KK, Krupanidhi SB (2020). Temperature dependent “S-shaped” photoluminescence behavior of InGaN nanolayers: optoelectronic implications in harsh environment. ACS Appl Nano Mater.

[43] Liu W, Zhao DG, Jiang DS, Chen P, Liu ZS, Zhu JJ, Shi M, Zhao DM, Li X, Liu JP, Zhang SM, Wang H, Yang H, Zhang YT, Du GT (2015). Temperature dependence of photoluminescence spectra for green light emission from InGaN/GaN multiple wells. Opt Express.

[44] Weng GE, Zhao WR, Chen SQ, Akiyama H, Li ZC, Liu JP, Zhang BP (2015). Strong localization effect and carrier relaxation dynamics in self-assembled InGaN quantum dots emitting in the green. Nanoscale Res Lett.

[45] Javier A, Magana D, Jennings T, Strouse GF (2003). Nanosecond exciton recombination dynamics in colloidal CdSe quantum dots under ambient conditions. Appl Phys Lett.

[46] Mehata MS, Ratnesh RK (2019). Luminescence properties and exciton dynamics of core–multi-shell semiconductor quantum dots leading to QLEDs. Dalton Trans.

[47] Hwang JI, Hashimoto R, Saito S, Nunoue S (2014). Development of InGaN-based red LED grown on (0001) polar surface. Appl Phys Express.

[48] Dussaigne A, Le Maitre P, Hass H, Pillet JC, Barbier F, Grenier A, Michit N, Jannaud A, Templier R, Vaufrey D (2021). Full InGaN red (625 nm) micro-LED (10 μm) demonstration on a relaxed pseudo-substrate. Appl Phys Express.

[49] Haemmer M, Roycroft B, Akhter M, Dinh DV, Quan Z, Zhao J, Parbrook PJ, Corbett B (2018). Size-dependent bandwidth of semipolar (11–22) light-emitting-diodes. IEEE Photon Technol Lett.

[50] Huang WT, Peng CY, Chiang H, Huang YM, Singh KJ, Lee WB, Chow CW, Chen SC, Kuo HC (2022). Toward high-bandwidth yellow-green micro-LEDs utilizing nanoporous distributed Bragg reflectors for visible light communication. Photonics Res.

[51] Huang Chen SW, Huang YM, Chang YH, Lin Y, Liou FJ, Hsu YC, Song J, Choi J, Chow CW, Lin CC, Horng RH, Chen Z, Han J, Wu T, Kuo HC (2020). High-bandwidth green semipolar (20–21) InGaN/GaN micro light-emitting diodes for visible light communication. ACS Photonics.

